# The effect of tight glycaemic control, during and after cardiac surgery, on patient mortality and morbidity: A systematic review and meta-analysis

**DOI:** 10.1186/1749-8090-6-3

**Published:** 2011-01-10

**Authors:** Kristin K Haga, Katie L McClymont, Scott Clarke, Rebecca S Grounds, Ka Ying B Ng, Daniel W Glyde, Robert J Loveless, Gordon H Carter, R Peter Alston

**Affiliations:** 1 School of Medicine and Veterinary Medicine, University of Edinburgh, Chancellors Building, 47 Little France Crescent, Edinburgh, EH16 4TJ, UK; 2 Department of Anaesthesia, Critical Care and Pain Medicine, School of Medicine and Veterinary Medicine, Royal Infirmary of Edinburgh, 51 Little France Crescent, Old Dalkeith Road, Edinburgh EH16 4SA, UK

## Abstract

**Background:**

Hyperglycaemia is a common occurrence during cardiac surgery, however, there remains some uncertainty surrounding the role of tight glycaemic control (blood glucose <180 mg/dL) during and/or after surgery. The aim of this study was to systematically review the literature to determine the effects of tight versus normal glycaemic control, during and after cardiac surgery, on measures of morbidity and mortality.

**Method:**

The literature was systematically reviewed, based on pre-determined search criteria, for clinical trials evaluating the effect of tight versus normal glycaemic control during and/or after cardiac surgery. Each paper was reviewed by two, independent reviewers and data extracted for statistical analysis. Data from identified studies was combined using meta-analysis (RevMan5^®^). The results are presented either as odds ratios (OR) or mean differences (MD) with 95% confidence intervals (CIs).

**Results:**

A total of seven randomised controlled trials (RCTs) were identified in the literature, although not all trials could be used in each analysis. Tight glycaemic control reduced the incidence of early mortality (death in ICU) (OR 0.52 [95% CI 0.30, 0.91]); of post-surgical atrial fibrillation (odds ratio (OR 0.76 [95%CI 0.58, 0.99]); the use of epicardial pacing (OR 0.28 [95%CI 0.15, 0.54]); the duration of mechanical ventilation (mean difference (MD) -3.69 [95% CI -3.85, -3.54]) and length of stay in the intensive care unit (ICU) (MD -0.57 [95%CI -0.60, -0.55]) days. Measures of the time spent on mechanical ventilation (I^2 ^94%) and time spent in ICU (I^2 ^99%) both had high degrees of heterogeneity in the data.

**Conclusion:**

The results from this study suggest that there may be some benefit to tight glycaemic control during and after cardiac surgery. However, due to the limited number of studies available and the significant variability in glucose levels; period of control; and the reporting of outcome measures, further research needs to be done to provide a definitive answer on the benefits of tight glycaemic control for cardiac surgery patients.

## Background

Hyperglycaemia (defined as a blood sugar > 180 mg/dL, for the purpose of this review) during and after cardiac surgery is a well-documented phenomenon [1,2] and is part of the body's stress response to surgery, resulting in increased gluconeogenesis and glycogenolysis [3]. Uncontrolled hyperglycaemia can lead to: hypokalaemia, hyponatraemia, arrhythmias and an increased risk of ischemic brain injury [4]. In addition, it has been demonstrated that hyperglycaemia may predispose patients to an increased risk of post-surgical infections through impaired phagocytic activity and decreased neutrophil function [5,6]. However, attempts to control blood glucose levels by, intensive insulin treatment (IIT), runs the risk of hypoglycaemia with serious cardiovascular and neurological consequences.

Previously published evidence suggests that "tight" glycaemic control (defined as blood glucose maintained at < 180 mg/dL) in critically ill, surgical and non-surgical patients, improves morbidity and mortality [7], [8]. Based on these findings, the European Society of Cardiology recently issued guidelines [9] pertaining to the control of hyperglycaemia in diabetic patients in an Intensive Care Setting (ICU). However, the results of a recently conducted, large, randomised controlled trial, the NICE-SUGAR study, indicate that glycaemic control, below 108 mg/dL may actually increase the rate of all-cause mortality in ICU patients, both surgical and non-surgical [10].

When it comes to the role of tight glycaemic control, during and after cardiac surgery, there remains a certain degree of uncertainty. The purpose of this study was to conduct a systematic review of the literature on the effects of "tight" versus "normal" glycaemic control, peri and post-operatively, in patients undergoing cardiac surgery in an attempt to help clarify the potential benefits and risks associated with glucose control in this patient population.

## Methods

### Search Strategy

A comprehensive search of published, peer reviewed research was performed using Medline, the Cochrane Library, Embase, Ovid, NHS Scotland E-Library, SIGN (Scottish Intercollegiate Guidelines Network) and NICE (National Institute of Clinical Excellence) to identify relevant studies. The following exploded search terms: *'tight'*, *'glucose'*, *'control'*, *'cardiac'*, *'surgery'*, *'CPB'*, *'CABG'*, *'heart*,' '*intensive'*, *'aggressive'*, *'insulin'*, *'therapy' *and *'strict' *were used. The reference lists of relevant papers were then hand searched to identify any additional articles. Our search was limited to articles published in the English language.

### Study Selection Criteria

Each abstract returned using the above criteria was independently reviewed twice. Those studies identified as being RCTs were evaluated based on explicit inclusion and exclusion criteria. Studies were included which (1) reported data on cardiac-surgery patients; (2) compared patients whose blood glucose was controlled within a pre-determined, defined upper limit (tight control) with those patients whose blood glucose levels were either uncontrolled or maintained below a higher limit (normal control); (3) that presented original study data, or data that was extracted from a larger systematic review, and (4) showed random allocation of patients to the tight or normal blood glucose control groups. Studies were excluded which (1) involved non-cardiac surgery patients, (2) only evaluated the method of glucose control, rather than outcomes, (3) had no extractable data (e.g. no means, standard deviations or reference to the percentage of patients with an adverse outcome).

### Outcome measures

The outcome measures were chosen on the basis that at least three independent sources reported extractable data for patients with and without tight glycaemic control. The following outcome measures were reviewed: (1)early mortality (within the first 30 days after surgery or mortality in Coronary Care Unit (CCU)/ICU); (2) atrial fibrillation (AF); (3) time in ICU; (4) time on mechanical ventilation; (5) need for epicardial pacing.

### Meta-Analysis

The meta-analysis was performed using RevMan5^® ^software, from the Cochrane Collaboration. The incidences of adverse events are presented as odds ratios (OR), and results for continuous measures were presented as mean differences (MD). A 95% confidence interval (CI) was used, and the probability for overall effect was deemed significant if p < 0.05. Heterogeneity values (I^2^) are also reported for each of the outcome measures.

## Results

### Search Results

**Figure 1:**
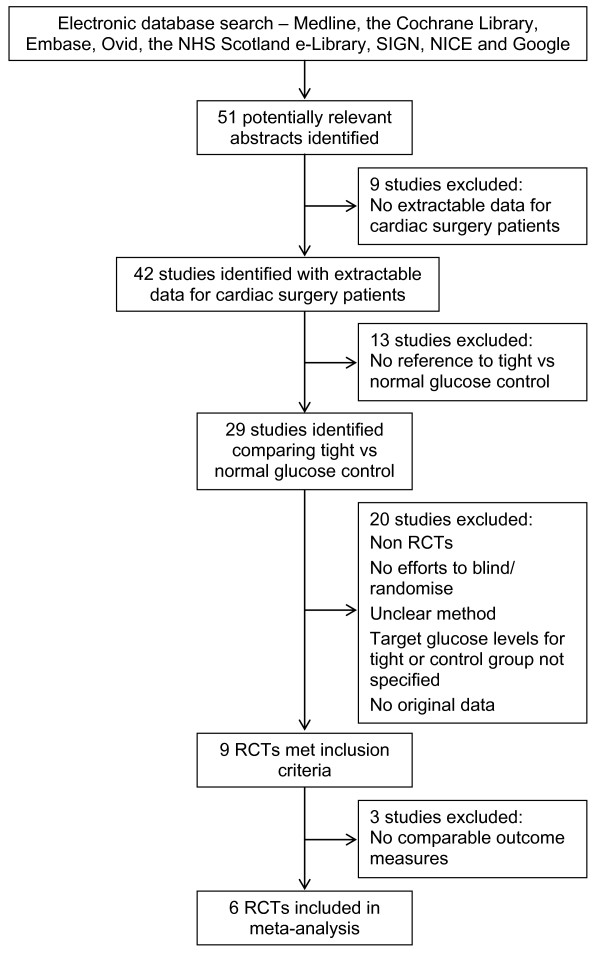
**Flow chart of study identification, inclusion and exclusion criteria**. This flow diagram illustrates the databases searched in this review, the resulting number of potential studies identified by this search; and the number and reasons for excluding studies based our pre-determined criteria. Following this process, seven RCTs were identified as meeting all criteria and were included in the meta-analysis. The two studies that were excluded at the end of the process presented only data on inflammatory markers and cytokines rather than clinical outcomes or endpoints.

The results of the literature search identified 51 potentially relevant reports on glucose control in cardiac surgery (Figure 1). Based on the inclusion and exclusion criteria, only nine of these studies met the criteria as being randomised controlled trials. In these studies, the definition of "tight" glycaemic control varied significantly, with upper limits ranging from 100 mg/dL to 200 mg/dL. Conversely, "normal" control was defined as either upper limit of 180 mg/dL to 250 mg/dL or no active glycaemic control [11-18]. Of these, two studies had to be excluded [5,11] as the only outcome measures they reported were the effects of glycaemic control on biochemical markers of inflammation, and thus were not combinable with the other identified studies. Two of the studies contained both diabetic and non-diabetic patients, with random allocation to treatment groups [13,15]. One study included only diabetic patients [18]. The remainder of the studies contained only non-diabetic patients. Table 1 summarises the seven RCTs included in the meta-analysis.

**Table 1:** Table of included RCTs

		Subjects (n)		Glucose ranges		Early Mortality			AF			Pacing			Time in ICU*			Time on Ventilation		
Author (year)	Control Period	Total (non-DM/DM)	Tight/normal control	mg/dL		n(%)		*p*	n (%)		*p*	n (%)		*p*	Mean ± SD		*p*	Mean ± SD		*p*
				Control	Tight	Control	Tight		Control	Tight		Control	Tight		Control	Tight		Control	Tight	
Ingels [15] (2006)	Post	970 (N/A)	477/493	< 220	< 110	37%	16%	0.005	N/A	N/A	--	N/A	N/A	--	N/A	N/A	--	N/A	N/A	--

Chaney [12] (1999)	Peri	20 (20/0)	10/10	-	100 - 150	N/A	N/A	--	2 (20%)	4 (40%)	N/A	N/A	N/A	--	N/A	N/A	--	N/A	N/A	--

Gandhi [13] (2007)	Peri	400 (327/73)	185/186	< 200	< 100	N/A	N/A	--	59 (32%)	54 (29%)	n.s.	N/A	N/A	--	N/A	N/A	--	N/A	N/A	--

Hoedemaekers [14] (2005)	Post	20 (20/0)	10/10	< 200	80 - 110	N/A	N/A	--	N/A	N/A	--	N/A	N/A	--	20.3 ± 2.5	22.1 ± 1.8	0.09	9.8 ± 4.6	11.2 ± 6.6	n.s.

Groban [16] (2002)	Peri/Post	381 (381/0)	188/193	-	80 - 120	0%	1.60%	n.s.	68 (35%)	60 (32%)	n.s.	2 (1%)	3 (2%)	n.s.	N/A	N/A	--	32 ± 4.0	31 ± 4.0	N/A

Koskenkari [17] (2005)	Peri	40 (40/0)	20/20	< 180	108 - 180	N/A	N/A	--	14 (70%)	12 (60%)	N/A	12 (60%)	3 (15%)	0.008	48 ± 28.8	76.8 ± 112.8	0.8	12.9 ± 14.2	11.4 ± 20.1	0.1

Lazar [18] (2004)	Peri	141 (N/A)	72/69	< 250	125 - 200	0%	0%	n.s.	29 (42%)	12 16.6%	0.002	27 (39%)	10 13.8%	0.001	32.8 +2.6	17.3 +1.0	0.001	10.7 ± 0.6	6.9 ± 0.3	< .001

This table describes the seven RCTs that met our inclusion/exclusion criteria and were included in the meta-analysis. In the table, "peri" and "post" designate the timing of glycaemic control as either during surgery (peri) or after surgery (post). One study, Groban et al, maintained tight glycaemic control both during and after surgery. "DM" = diabetes mellitus (not specified if this was Type I or Type II). The glucose ranges for the "tight" control and "normal" control groups are given. Where no glucose range is listed in the "normal" ranges indicates that no attempt was made to keep the patients under any glycaemic control. The number of cases (n%) or the mean ± SD for each of the five outcome measures is listed for each study. Not every RCT included provided data on each of the outcome measures, thus some boxes are blank or listed as "N/A" (not available). Where provided, the p-value for the statistical comparison within that study is also listed in the table.

### Meta-Analysis Results

#### Early Mortality

**Figure 2:**
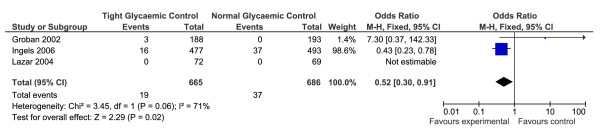
**Results of the meta-analysis performed on the incidence of early mortality, following cardiac surgery, for patients with and without tight glycaemic control**. This figure illustrates the forest plot created as a result of the meta-analysis for early mortality data. "Early mortality" was defined as death in CCU or within 30 days. Only three of the seven studies presented useable data and were included in the analysis. Tight glycaemic control peri- and post-operatively significantly reduced early mortality as compared to normal glycaemic control (p = 0.02).

Three RCTs were identified that cited "early" mortality as a primary outcome [15,16,18] (n = 1492). For the purpose of our analysis, "early" mortality was defined as mortality within the first 30 days [18], or mortality in CCU/ICU [15,16]. Tight glycaemic control significantly reduced early mortality following cardiac surgery (OR = 0.52, 95% CI 0.30 to -0.91, p < 0.02, Z = 2.29, heterogeneity I^2 ^= 71%, p < 0.06). Only two of the studies actually reported patient mortality events [16,15](Figure 2). The Lazar [18] study reported zero mortality in both their tight and normal control groups, however, the study comprised relatively small numbers (overall n = 141) when compared to the other two.

#### Atrial Fibrillation

**Figure 3:**
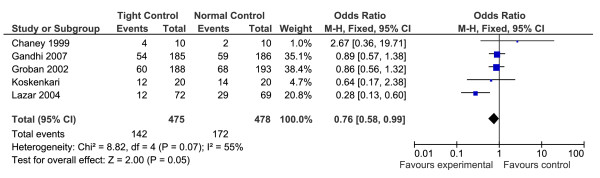
**Results of the meta-analysis on the incidence of atrial fibrillation following cardiac surgery, for patients with and without tight glycaemic control**. This figure illustrates the forest plot created as a result of the meta-analysis performed on the incidence of atrial fibrillation (AF) following cardiac surgery for the tight and normal glycaemic control groups. As can be seen, tight glycaemic control demonstrated a borderline significant reduction in the incidence of AF following cardiac surgery (p = 0.05). Only one study, Chaney et al reported a higher incidence of AF in patients in the tight glycaemic control group, however their overall patient numbers was extremely small (n = 20).

The occurrence of atrial fibrillation (AF), post cardiac surgery, was reported in five of the RCTs (total n = 488) [12,13,16-18]. The results of the meta-analysis revealed a significant reduction in AF in patients with tight glycaemic control during surgery (OR = 0.76, 95% CI 0.58-0.99, p = 0.05, Z = 2.00, heterogeneity I^2 ^= 55%, p = 0.07) (Figure 3).

#### Length of Time in ICU/CCU

**Figure 4:**
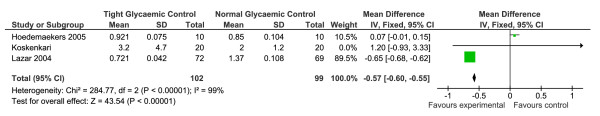
**Results of the meta-analysis conducted on the time spent in CCU/ICU, for patients with and without tight glycaemic control, following cardiac surgery**. This figure illustrates the forest plot created from the meta-analysis of total time spent in CCU or ICU following cardiac surgery, for those patients with and without tight glycaemic control. Although the results suggest that patients who were randomised to the tight glycaemic control group spent significantly less time in CCU/ICU (p < 0.00001), the significant heterogeneity (99%) of this sample makes it difficult to interpret these results. The data are significantly weighted by one study, the Lazar (2004) study, and the times spent in CCU/ICU vary dramatically between groups.

The duration of time spent in ICU/CCU following cardiac surgery, for patients with and without tight glycaemic control, was reported in five of the RCTs [13-15,17,18] (n = 201). However, two of these studies presented their results in median number of days [14,13], and three of them presented their data as mean hours or days. We wrote to the authors of the two papers that presented their data as a median and range, and asked if they could provide their data as means and standard deviations, unfortunately, these data were not available. Therefore, we conducted a meta-analysis of the three papers that present their data as means and SDs [14,17,18]. The results of this analysis are shown in Figure 4. There was a significant effect of tight glycaemic control on reducing the time spent in ICU (OR = -0.57, 95% CI -0.60 to -0.55, p < 0.00001, Z = 43.54, heterogeneity I^2 ^= 99%, p < 0.00001). The results of this analysis are heavily weighted by one study, Lazar et al [18], and the mean values differ greatly between Lazar et al's [18], Hoedemaekers et al's [14], and Kosenkari et al's [17] studies, which is reflected in the heterogeneity analysis.

#### Length of Time on Mechanical Ventilation

**Figure 5:**
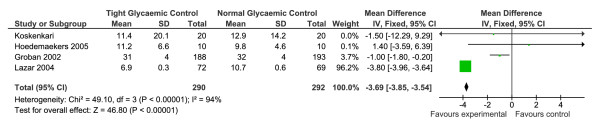
**Results of the meta-analysis conducted on the time spent on mechanical ventilation, following cardiac surgery, for patients with and without tight glycaemic control**. This figure illustrates the forest plot created from the meta-analysis of the time spent on mechanical ventilation, following cardiac surgery, for patients with and without tight glycaemic control. The results of this analysis suggest that patients who experienced tight glycaemic control peri and/or post-operatively spent significantly less time on mechanical ventilation (p < 0.00001). However, the results are heavily weighted by the Lazar (2004) study due to the wide ranges of time and large standard deviations in the other four studies.

There were five RCTs that examined the time spent on mechanical ventilation following cardiac surgery, with and without tight glycaemic control [13,14,16-18]. One study was excluded as they presented their data as the number of patients who were delayed in being removed from ventilation [13], rather than as the mean number of hours on ventilation. The four studies that presented their data as mean hours on ventilation were compared in a meta-analysis [14,16-18] (n = 582), and the results are shown in Figure 5. Overall, there was a significant reduction in the amount of time spent on ventilation for those patients who had tight glycaemic control compared to controls however, there was also significant heterogeneity in the data which were heavily weighted by the Lazar [18] study (OR = -3.69, 95% CI -3.85 to -3.54, p < 0.000001, Z = 46.80, heterogeneity I^2 ^= 94%, p < 0.00001).

#### The Need for Epicardial Pacing

**Figure 6:**
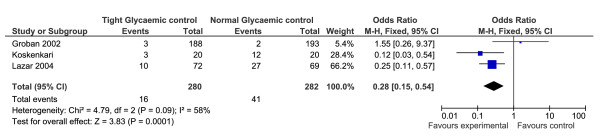
**Results of the meta-analysis conducted on the need for epicardial pacing, following cardiac surgery, in patients with and without tight glycaemic control**. This figure illustrates the forest plot produced as a result of the meta-analysis on the need for epicardial pacing in patients with and without tight glycaemic control. As can be seen in the figure, those patients with tight control experienced less need for epicardial pacing (p = 0.0001).

The need for epicardial pacing following surgery was recorded as an outcome measure in three of the RCTs [16-18] (n = 562). Two of the three studies reported a reduced need for pacing in patients with tight glycaemic control. A meta-analysis performed on these, three studies, revealed a significant effect of tight glycaemic control on the need for epicardial pacing, with those in the tight control group requiring less pacing (OR = 0.28, 95% CI 0.15-0.54, p = 0 < 0.001, Z = 3.83, heterogeneity I^2 ^= 58%, p = 0.09) (Figure 6).

## Discussion

To our knowledge, this is the first systematic review and meta-analysis conducted solely on the effects of tight and conventional glycaemic control in patients undergoing cardiac surgery. In our original literature serach, we did identify three systematic reviews exploring the effect of insulin infusion in critically ill patients (both medical and surgical) [19-21]. However, in these reviews, only one analysed the data for cardiac surgery patients independently [21] and in these a targeted glucose level was not part of the infusion protocol and thus a comparison of "tight" versus "normal" control cannot be made. However, in this review [21] there was no significant effect of insulin (normally GIK - glucose, insuling, potassium) infusion on mortality. The results from this review indicate that tight glycaemic control, both peri and post-operatively, may reduce mortality and morbidity in cardiac surgery patients.

### The effect of tight glycaemic control on mortality following cardiac surgery

The results of our meta-analysis on early mortality, including data for almost 1500 patients, from three RCTs, suggest that there may be a significant reduction in early mortality in the tight glycaemic control groups. However, this data was heavily weighted by the Ingels et al study [15], while the other two studies both had very low patient numbers and low mortality events in their data [16,18]. In a previous review by Gandhi et al [19] they reported mortality data for 4355 patients, receiving insulin infusions during and/or after surgery. Although they found a significant reduction in overall mortality with the use of insulin, they stated that the cumulative evidence reported in their study is still insufficient, as a power calculation performed based on a 25% risk reduction with 90% power, taken at the 0.05, two-sided significance level, would require over 11,000 patients in order to be adequately powered. It is clear from this that our results, based on less than 1500 patients, are equally underpowered to answer this question conclusively and further large-scale trials, with appropriate double-blinding and definable outcome measures are still needed.

Of interest, is the possible effect that the varying duration of glycaemic control may have had on data in this review. In the Groban study [16], where there was very little reported early mortality, tight glycaemic control was maintained in both the peri- and post-operative periods. Whereas the Ingels study [15], with the highest rate of overall early mortality (in both treatment groups), only reported controlling glucose levels post-operatively. It is not possible to comment on the effect which the differences in the timing of glycaemic control may have had on the results. However, it does appear that tight control extended beyond the peri-operative period may reduce the incidence of early mortality. Previous reviews on the effect insulin infusion and patient mortality have had similar mixes in the data, with infusion times being variable between studies. In the future, research is needed to clearly define the potential benefit of glycaemic control peri operatively vs post-operatively vs a combined method in order to tease out the effect of timing on outcome.

Only one study, Ingels et al [15], looked at long-term mortality as a consequence of tight or normal glycaemic control, and found no significant differences between the groups at two to four years following surgery. This suggests that tight compared to normal glycaemic control may have a short-term effect on reducing mortality, but this difference is not translated into a long-term survival.

Our results on the effects of tight glycaemic control on mortality are in contrast to those reported in the NICE-SUGAR trial [10], which found that blood glucose control below 108 mg/dL was associated with a significant increase in all-cause mortality in ICU patients. The reason for the discrepancy may be due to one or more of the following reasons; 1) The NICE study only looked at glucose control in the post-operative period. The studies in this review include data from both the peri-operative and post-operative periods; 2) The NICE study included data from surgical and non-surgical patients, where this review includes data only from cardiac surgery patients, who may have a different response to tight glycaemic control compared to the general ICU patient; and 3) the definition of "tight" glycaemic control in the NICE study was 80-108 mg./dL, whereas in this review, the glycaemic ranges used varied from < 110 mg/dL [15], 80-120 mg/dL [16] and 125-200 mg/dL [18]. Interestingly, the only study that did not report ANY mortality, the Lazar study, also had the highest range of blood glucose levels in their tight control group (125-200 mg/dL). This raises the question as to the optimum level of glucose control, which has yet to be evaluated in a randomised trial, and the possibility that lower isn't necessarily better.

### Incidence of atrial fibrillation and the need for epicardial pacing

Atrial fibrillation (AF) following cardiac surgery is common [22,23], affecting up to 20-30% of patients [23] and may increase surgical morbidity and mortality [24]. In our review, tight glycaemic control demonstrated a borderline significant reduction in the incidence of AF following cardiac surgery (p = 0.05), although four of the five RCTs reported fewer AF episodes in their tight control group when compared to their normal glucose control group. There was a stronger effect on the reduced need for epicardial pacing (p = 0.0001) in patients with tight glycaemic control during cardiac surgery. However, epicardial pacing is used not only to control episodes of AF and other supra-ventricular tachycardias, but also ventricular tachycardias and possibly bradycardias, therefore the reduced need for pacing cannot be interpreted solely as a reduced incidence of AF.

The study by Groban et al [16] also examined the need for cardioversion and/or medication to reverse AF following a coronary artery bypass graft. Although fewer of the patients with tight glycaemic control experienced AF, more of them actually needed cardioversion for these AF episodes, however this was not statistically significant (p = 0.40). Also in this study, patients in the treatment group received more anti-arrhythmic medications post-surgery, which could then affect the number of AF episodes reported. Unfortunately, the study does not detail if these anti-arrhythmic medications were given as prophylaxis or whether they were given solely in response to AF episodes. None of the other studies in this meta-analysis gave any details on the need for cardioversion or the use of anti-arrhythmic medications.

The pathological basis behind the increased incidence of AF following cardiac surgery is unclear. Previous studies have suggested that AF may be due to the effects of surgical trauma [25], inadequate cooling of the atrium [26,27], withdrawal of pre-operative beta-blockers [28] or possibly increased sympathetic activity [29]. Consequently, there is a change or dispersion in the local refractory periods in the atria, predisposing the tissue to the development of fibrillation.

Due to the lack of understanding of the basic mechanisms of AF following cardiac surgery, it is difficult to propose a theory on the protective role of tight glycaemic control. However, research has shown that insulin acts on myocytes to increase their uptake of potassium [30], which may then lead to a recovery of sinus rhythm following surgery. Zhang and colleagues have indicated that insulin may act to stabilise the myocyte membrane potential through the discovery of a new ion channel which is sensitive to insulin [31]. In order to evaluate this fully, it would be necessary to compare both patients' glycaemic control during surgery and the amount of insulin they received. Unfortunately, this data was not available from the studies included in this review.

### Time spent on mechanical ventilation and in CCU/ICU following cardiac surgery

The results of this review on the length of time patients spent on mechanical ventilation and overall time spent in CCU or ICU are more difficult to interpret. In both instances, the results are significant, but heavily weighted by Lazar et al's study [18]. The only conclusions that can be drawn from this data is that tight glycaemic control may equate to better overall recovery, however, there needs to be more research done in this area, as well as some discussion of the mechanism by which glycaemic control may exert these effects, as these are not considered by the authors from the original papers. Some of these mechanisms may include (1) altering the immune response and reducing the risk of infection [5,32,33]; (2) avoiding the pro-inflammatory effect of hyperglycaemia which may contribute to post-operative capillary leak syndrome, platelet dysfunction and a higher risk of post-operative complications [34,35]; (3) The insulin administration may protect the heart in ischaemic conditions by increasing glucose uptake by myocytes, increasing glycogenesis and reducing the concentration of free fatty acids [36,37].

### Limitations of this review

Although the results from this review raise the possibility that tight glycaemic control may confer some benefit to patients undergoing cardiac surgery, there are a number of substantial limitations that must be considered when interpreting or applying these results. Firstly, there were few eligible RCTs with comparable outcomes that we could include in this review. The trials which we did identify and were able to include used relatively small patient populations as well as poorly defined outcome measures, causing heterogeneity to be a persistent problem in our meta-analysis. A number of outcomes included in our results were secondary outcome measures and therefore the original RCT study design may not have been optimised for the evaluation of those outcomes.

In addition, there were important methodological differences between studies: the proportion of diabetic and non-diabetic patients; definitions of "tight" and "normal" glycaemic control during and/or after surgery; and the method of measuring the outcome measures. We were unable to compare some data, despite contacting the authors, because of different approaches to collecting and reporting information, for example, the method of measuring time spent in ICU.

## Conclusion

The results of this systematic review of the literature and meta-analysis indicate that tight compared to normal glycaemic control during and after cardiac surgery may impart some benefit to patients following cardiac surgery, including a reduction in early mortality and the incidences of post-operative AF and the need for epicardial pacing. There is some evidence that tight glycaemic control may also improve overall recovery by reducing the time spent on mechanical ventilation and the time in CCU/ICU. However, these results should be interpreted with caution due to the high levels of heterogeneity in the meta-analysis. Additional research is needed in order to provide more definitive answers on the potential benefit of tight glycaemic control during cardiac surgery. In addition, future research should (1) address the differences between diabetic and non-diabetic patients with respect to tight control and outcome; (2) the need to have a clearly defined and accepted glycaemic range that is considered "tight" control versus "normal" control; (3) include larger, randomised, blinded studies; and (4) examine longer term outcomes in addition to those immediately after surgery. The idea that some glycaemic control is needed during major surgery such as coronary artery bypass grafting, is currently well accepted, but further research is now required to determine the precise range to confer the most benefit, possibly by allocating patients to groups with increasing levels of tight glycaemic control. The timing of this control and the benefits, risks and underlying physiological mechanisms associated with aggressive glycaemic control also require further investigation.

## List of Abbreviations

**AF**: atrial fibrillation; **CCU**: coronary care unit; **CI**: confidence interval; **GIK**: glucose/insulin/potassium; **ICU**: intensive care unit; **IIT**: intensive insulin treatment; **MD**: mean difference; **NICE**: National Institute of Clinical Excellence; **OR**: odds Ratio; **SIGN**: Scottish Intercollegiate Guidance Network; **RCT**: randomised controlled trial

## Statement of Competing Interests

The authors declare that they have no competing interests.

## Authors' contributions

KKH literature searching, data extraction, statistical analysis and interpretation, drafting of original document, editing of document. KM literature searching, data extraction, drafting of original document, editing of document. SC literature searching, data extraction, drafting of original document, editing of document, computer support. RSG literature searching, data extraction, editing/revision of document. KYN literature searching, data extraction, drafting of original document, editing/revision of document. DWG literature searching, data extraction, editing/revision of document. RL literature searching, data extraction, editing/revision of document. GC literature searching, data extraction, editing/revision of document. RPA data extraction and interpretation; statistical analysis/interpretation, drafting of original document, editing/revision of document, study supervision.

**All authors have read and approved the final manuscript**.

## Information about the Authors

KKH: is a graduate medical student (4^th ^year), studying at the University of Edinburgh. She has over 9 years of post-doctoral research experience and has published previous systematic reviews. KM, RSG, KYN, DWG, RL and GC are all fourth year medical students, who conducted this study as part of a student-selected research module at the University of Edinburgh. RPA is a consultant anaesthetist at the Royal Infirmary of Edinburgh, and supervised the project conducted by the students.
